# An analysis of the titles of papers submitted to the UK REF in 2014: authors, disciplines, and stylistic details

**DOI:** 10.1007/s11192-016-2081-4

**Published:** 2016-07-29

**Authors:** John Hudson

**Affiliations:** Department of Economics, University of Bath, Bath, BA2 7AY UK

**Keywords:** Multiple authors, Journal title length, REF, Colon, Citations, Question marks, 01A80, 62J99, A12, B40

## Abstract

In 2014 over 52,000 academics submitted >155,500 journal articles in 36 different disciplines for assessment in the UK’s four-year Research Evaluation Framework (the REF). In this paper the characteristics of the titles of these papers are assessed. Although these varied considerably between the disciplines, the main findings were that: (i) the lengths of the titles increased with the number of authors in almost all disciplines, (ii) the use of colons and question marks tended to decline with increasing author numbers—although there were a few disciplines, such as economics, where the reverse was evident, (iii) papers published later on in the 4-year period tended to have more authors than those published earlier, and (iv), in some disciplines, the numbers of subsequent citations to papers were higher when the titles were shorter and when they employed colons but lower when they used question marks.

## Introduction

Authors of an academic journal paper tend to choose a title with a view to maximising the impact of that paper, particularly perhaps in terms of citations. Hartley (2008) lists thirteen different types of title, including those that state findings and those that ‘bid for attention’. There are two factors to consider here, the information content of the title, and the extent to which it attracts academic attention. For example, longer titles may increase information content up to a certain point but reduce the interest generated. Use of colons may facilitate the latter, particularly if part of the split title is focused on generating interest. It may also facilitate a title which is more informative, or enables the authors to strike a better balance between the twin goals of the title. But in the process, the title tends to become both longer and more complex (van Wesel et al. 2014) and in some, but not all, circumstances this may lead authors to shorten the title and in the process reduce its readability and attractiveness.^1^ Ball (2009) argues that question marks can be used to both inform and stimulate interest. However they set a certain style to the title and in this way may also constrain it. For example we later provide evidence that question mark based titles are significantly longer than other titles. However, a further factor complicates this decision and that relates to when there is more than one author to the paper. In that case the title may be a compromise between what the various authors would wish, and may be sub-optimal to all of their preferences.

In this paper we examine the impact of multiple authorship on various aspects of the title and thence their impact on citations. There has been some work on different aspects of the title including the impact of multiple authorship, but this has tended to be on particular academic disciplines or groups of disciplines, rather than across all disciplines as a whole. Our paper looks at journal papers across disciplines making use of a unique data base from the UK. Being as the UK has academics from all over the world who work with other academics from across the world, it does not reflect the practices of UK academics as such, but relates to papers most of whose authors work in the UK. Apart from exploring aspects such as title length and the use of colons and question marks, we will also be implicitly examining one further aspect of academic work, i.e. the amount of time it takes to complete a paper. We cannot do this directly in this research with the data base we have, although in future research that may be possible. Instead we will be analysing the issue indirectly via an observable variable which, given certain assumptions, is linked to research time.

The data is based on the Research Excellence Framework^2^ (REF) of 2014, which is the latest in a series of exercises seeking to evaluate the quality of research done in UK universities across a range of disciplines—termed units of assessment (UoAs). It replaced the previous exercise known as the Research Assessment Exercise (RAE). In the REF there were 36 such UoAs as listed in Table 1. Throughout the paper we refer to these by the shortened names in Table 2. Each entry to a UoA was scrutinised by a sub-panel working within the framework of four main panels A–D, as also listed in Table 1. The main scrutiny was on the quality of academic research contributions, primarily journal articles and books, which were individually assessed by panel members. Each individual within a submission was allowed to submit four contributions. Each submission was also accompanied by a research environment statement and a number of impact case studies which highlighted the impact of their research on society or the economy, widely defined. These were also evaluated and the overall grade given to the submission was a combination of the evaluations of all three. But the greatest weight was given to publications and it is these that we focus on in this paper.

**Table 1:** The panels and UoAs of the 2014 REF *Source* http://www.ref.ac.uk/media/ref/content/pub/panelcriteriaandworkingmethods/01_12.doc

Panel A	
1	Clinical medicine
2	Public health, health services and primary care
3	Allied health professions, dentistry, nursing and pharmacy
4	Psychology, psychiatry and neuroscience
5	Biological sciences
6	Agriculture, veterinary and food science
Panel B	
7	Earth systems and environmental sciences
8	Chemistry
9	Physics
10	Mathematical sciences
11	Computer science and informatics
12	Aeronautical, mechanical, chemical and manufacturing engineering
13	Electrical and electronic engineering, metallurgy and materials
14	Civil and construction engineering
15	General engineering
Panel C	
16	Architecture, built environment and planning
17	Geography, environmental studies and archaeology
18	Economics and econometrics
19	Business and management studies
20	Law
21	Politics and international studies
22	Social work and social policy
23	Sociology
24	Anthropology and development studies
25	Education
26	Sport and exercise sciences, leisure and tourism
Panel D	
27	Area studies
28	Modern languages and linguistics
29	English language and literature
30	History
31	Classics
32	Philosophy
33	Theology and religious studies
34	Art and design: history, practice and theory
35	Music, drama, dance and performing arts
36	Communication, cultural and media studies, library and information management

**Table 2:** Summary data on the UoA submissions

	Title length	Citations	Authors	Date	Colon %	Question mark %	% Journal papers
Panel A: means	*109.85*	*27.65*	*7.35*	*2010.53*	*26.04*	*3.19*	*99.63*
Clinical medicine	113	40.6	12.3	2010.47	19.68	1.04	99.90
Public health	117.1	37.1	11.6	2010.58	59.51	6.02	99.67
Allied health	109.3	14.9	6.7	2010.58	32.4	4.09	99.06
Psychology	97.3	25.1	6.4	2010.51	36.31	5.55	99.58
Biological sciences	99.9	30.2	7.6	2010.44	11.16	1.32	99.69
Agriculture	110.4	15	7.1	2010.55	18.46	2.29	99.23
Panel B: means	*88.50*	*23.60*	*5.00*	*2010.47*	*14.24*	*1.00*	*99.09*
Earth systems	96.8	23.6	6.5	2010.35	26.07	3.62	99.09
Chemistry	98.7	26	6.1	2010.38	22.88	1.19	99.83
Physics	80.2	40.4	131.2	2010.2	14.24	1.46	99.15
Mathematical sciences	69.2	na	2.8	2010.62	9.37	0.68	96.31
Computer science	72.9	11	3.8	2010.58	18.52	1.00	78.81
Aeronautical engineering	93	3.4	4.2	2010.47	13.34	0.80	99.30
Electrical engineering	88.4	25.3	5	2010.43	10.2	0.50	99.18
Civil engineering	88.5	na	3.7	2010.57	19.16	2.15	97.69
General engineering	90.6	13	7.9	2010.48	13.76	0.69	98.67
Panel C: means	*86.00*	*6.30*	*2.40*	*2010.80*	*54.55*	*11.81*	*77.89*
Architecture	87.3	4.8	2.8	2010.84	46.45	7.64	77.89
Geography	94.3	13.1	4.4	2010.81	47.09	7.35	82.73
Economics	66	6.3	2.3	2010.73	30.55	9.46	92.27
Business	83.7	9.1	2.7	2010.76	53.23	11.63	95.66
Law	79.9	na	1.3	2010.78	54.55	18.82	62.64
Politics	79.3	na	1.5	2010.86	60.14	19.20	70.63
Social work	91.7	6	2.5	2010.8	65.42	15.94	77.38
Sociology	81.6	na	1.9	2010.77	66.05	12.88	76.16
Anthropology	86	na	2.4	2010.77	60.59	11.81	67.31
Education	92.8	4.2	2.4	2010.83	61.17	13.29	78.40
Sport, leisure and tourism	97.4	33.8	4.4	2010.84	34.52	6.33	96.77
Panel D: means	*77.65*	*10.00*	*1.30*	*2010.85*	*61.29*	*7.81*	*40.64*
Area studies	83.8	2	1.5	2010.74	63.93	12.40	56.61
Modern languages	82.1	27	2.3	2010.84	59.96	7.60	48.56
English language	74.7	na	1.2	2010.9	66.03	5.12	35.81
History	85.4	na	1.1	2010.81	62.62	8.03	44.18
Classics	69.7	na	1.2	2010.92	49.75	6.72	29.00
Philosophy	51.1	na	1.1	2010.95	3.29	9.23	61.85
Theology	77.3	na	1.1	2010.78	57.44	10.21	37.10
Art and design	76.4	na	2.1	2010.86	55.58	4.51	26.64
Music and drama	78	na	1.4	2010.94	65.24	6.64	29.82
Media studies	82.2	10	1.8	2010.8	65.75	9.76	52.72

Title length is measured by the number of characters including spaces. For the first four columns, the data relating to each UoA reflects the mean in the UoA. The Panel values are the medians of the UoA numbers

These papers allow us to evaluate the working habits of academics in different disciplines, with the analysis focusing on the best work in these disciplines because this work was chosen to submit to the REF. In doing this we shall be analysing the average number of authors, the length of titles, the use of colons and question marks, and the number of citations. We will also examine the impact of multiple authorship on the working style of academics and the number of citations each paper gets. Being as our analysis is across disciplines, it helps explain some of the contradictory conclusions previously reported in the literature which tended to be focused on a limited number of disciplines. Finally, the analysis will shed light on the weaknesses of the REF in evaluating academic quality within and between disciplines.

The paper proceeds as follows. In the next section we shall review the relevant literature. We then discuss the data and how they were collected and analysed. Surprisingly perhaps, this was not totally straightforward. We then present the results, both summary data and regression results. Much of the work in this area tends to simply report, for example, average citations or incidence of papers with and without colons. But this might lead to a spurious correlation if title length, or the number of authors, impacts on citations, and use of colons is correlated with either of those two variables. In order to unwind multiple impacts, one needs multiple regression analysis. This we use both to analyse (i) the impact of author numbers on title length, use of colons and question marks and (ii) the impact of these title characteristics on subsequent citations. We will also analyse the impact of multiple authorship on when the paper was published within the context of the REF cycle. Finally, we conclude the paper.

## Literature and expectations

There has been substantial work done on the characteristics of journal titles in different disciplines. Titles serve at least two purposes (Lewison and Hartley 2005). Firstly they need to attract readers. Secondly they need to inform the reader about the paper’s contents. The structure of the title can do this in several ways. Lewison and Hartley argued that a two part structure using a colon both increases the number of words and increases the information content. There are substantial disciplinary differences in the use of colons. For example, usage tends to be more common in psychology (50 %) than computer science (7 %) (Lewison and Hartley 2005). In contrast, they concluded that authors rarely used a question mark in their titles. However, Ball (2009) found the use of a question mark in a title to have increased over time and to be higher in medicine (approx. 5 %) than life sciences (approx. 2.3 %) which in turn was higher than physics (approx. 0.57 %).^3^ Ball goes on to argue that the use of question marks can be explained as they both help to structure the title in a way as to provide informational content, but they also may stimulate interest in the paper, by provoking a potential reader. He also argues that they may also facilitate the rapid publication of results about which the authors are not fully confident (Ball 2009, p. 677). In addition there is a trend for longer titles (White and Hernandez 1991). There is also evidence to link the structure and style of the title to the number of authors, which in itself has tended to increase over time (Hudson 1996). Nagano (2015) argues that title lengths differ between disciplines, due in part to custom. She also finds titles tend to be longer in what she terms the ‘hard sciences’ such as medicine than the ‘soft sciences’ such as sociology. Finally, there is evidence of a positive relationship between the number of authors and the length of a paper’s title (White 1991), with this being more common among science, than social science or humanities, journals (Yitzhaki 1994). Lewison and Hartley (2005), as well as Hartley (2007), also find that in most disciplines single authors more commonly use colons in the title than is the case with multiple authors. However, when the number of co-authors is high this result tends to be reversed. This is in part consistent with the need for multiple authors to negotiate differences of opinion to a consensus (Johnson 1998). Johnson goes on to comment that co-authorship involves disinvesting in preferred ways of writing and this relates to language and not just content.

In persuading academics to read a paper, devices such as informative titles, question marks and colons may also stimulate impact as, for example, measured by the number of citations (Haslam et al. 2008). However, the relevant empirical work is not conclusive. Haslam et al., themselves found no link between ‘the catchiness’ of a title and citations in social and personality psychology, a similar finding to Hartley (2007) more generally. Indeed Hartley went on to comment that it would be startling if something so simple as a colon in a title led to repeated citations. Nonetheless, in a regression analysis Haslam et al. (2008) did find that a colon had a positive effect on citations, and title length had a small negative effect. Jacques and Sebire (2010) found a positive correlation equal to 0.62 which was significant at the 1 % level, between the number of citations and the length of the title. The presence of a colon also increased citations. However seeming to contradict this, Jamali and Nikzad (2011) detected a negative, although insignificant, correlation between citations and title length. But articles with a colon in the title received significantly, at the 5 % level of significance, fewer citations in six PLoS journals. Van Wesel et al. (2014) find citations to decline with title length and increase with the number of authors in a range of disciplines. They also increase with the use of a colon in applied physics, decline in general and internal medicine and to have no impact in sociology. To some extent these differences between different studies may be explained by differences between disciplines.

It has also been argued (Glanzel and Thijs 2004) that citations increase with the number of authors in a number of disciplines, e.g. evolutionary psychology (Webster et al. 2009), biology and biochemistry, chemistry, mathematics and physics (Vieira and Gomes 2010) and information science and technology (Levitt and Thelwall 2009). However in clinical psychology, educational measurement, and management science, Smart and Bayer (1986) found little effect. Such an effect, if it exists, might be expected because multiple authorship may improve paper quality (Haslam et al. 2008). It may also expand the network of the authors (Frenken et al. 2005). There is also a greater potential for self-citation.

There is evidence that coauthorship increases the time it takes to write a paper, Hollis (2001) emphasising the increased amount of time spent on redrafting the paper into a mutually acceptable form. In addition, using panel data on 339 economists he finds a negative relationship between research output and teamwork. Bidault and Hildebrand (2014) also suggest that the costs may increase as the size of the team increases. This is consistent with a long established literature which argues for the decreasing returns to teamwork in general (Hackman 1990). Katz and Martin (1997) focus specifically on co-authorship and the extent to which it coincides with collaboration. They discuss both the costs and the benefits as they apply across disciplines in general. In terms of the former they first mention time costs, i.e. time spent in preparing a research proposal, carrying out the research and writing up the results. Time costs may be greater when travel is involved. With respect to writing up costs, it is emphasised that there may be disagreements over the results and their significance. Specifically they comment that “Differences of opinion are almost inevitable and time will be needed to resolve these amicably. Writing up results jointly may also take more time where there are disagreements over the findings and their significance, or over who should be included among the co-authors and in what order they should be listed” (p. 15). Given this, it seems likely that there will also be disagreement, and hence the need for compromise, about other aspects of the paper including the title. Katz and Martin further argue that these costs are likely to increase with the number of people involved. Cummings and Kiesler (2005) analyse the coordination costs with respect to researchers in multi-university collaborations, mainly in the sciences and engineering but also psychology and mathematics. They conclude that the more universities involved in a collaboration, the fewer will be the number of coordination activities and project outcomes.

There is also a literature on the impact exercises like the REF and the RAE have on academics, their mode of working and their publications (Butler and Spoelstra 2014). Much of this results from institutional pressure to be enterable. Thus Hodder and Hodder (2010), in a New Zealand context, note that in the run up to the end of the relevant assessment period, 2006, publications increased and then declined in the years immediately following. There was also a tendency towards more co-authorship. Harley (2000), in the context of the RAE, argues that the need to produce papers sufficient to count within the relevant time period may have a negative impact on academic work. This is intuitively plausible and consistent with economic theory which would suggest that the REF in changing the incentive structure facing individuals will also change their behaviour. With no RAE/REF constraints we assume the academic would seek to maximise the quality and impact of their publications. As is generally the case, they will tend to prefer publications now rather than later, i.e. future publications are discounted. Whether to publish a piece of work now or in the future is then a trade-off between the discount factor and potential improvements which could be made to the publication by devoting more time to it. The requirement by the REF that the number of publications in each round must be no less than four, constrains the optimisation problem. For academics who are close to the deadline and need additional publications, the Lagrangean multiplier pertaining to the current REF period constraint will be positive and this may lead to publications being brought forward. For other academics who already have the required number of papers, this Lagrangean multiplier will be zero as the constraint is nonbinding, but there may be value in delaying publication in order to help meet the constraints of the next REF. There is evidence for both of these possibilities in addition to the observations made by Harley (2000). Firstly, for example, with people rushing the publication of edited books in order to fit within the (RAE) timeframe (Thomas 2011) and trying to persuade journals to publish their paper rapidly (Wellington 2003). Secondly, the author of this paper has direct knowledge of academics who have delayed publication with a view to enhancing their future value in the academic labour market. We also have the example of a young researcher at Warwick who gives the following advice “if you know you won’t be submitted for the REF 2014, then it might be worth holding off from publishing that 4* piece of work until after November 2013!”^4^ In other words delay publication until the next REF period.

Based on this discussion, we have a number of hypotheses which will be tested by the data.

- Firstly, the number of authors will vary between the disciplines and be greater for the sciences, including health, than the social sciences and the arts and humanities. In part this is because of the teams involved in lab work and in part due to the need to collaborate on ‘big science’.
- Secondly, although we anticipate some differences between disciplines, we expect the character of the title to change as the number of authors increases. Specifically, we anticipate title length will increase with the number of authors, as they seek a compromise which meets all of their views and preferences.
- However, the use of colons and question marks which complicate the title is expected to decline with the number of authors as, with two parts of a title to agree upon, their use makes agreement more difficult.
- In terms of impact, we anticipate that citations will increase with the number of authors, but decline with the length of the title, as too long a title makes it more difficult to digest and may reduce the attraction factor.
- Finally we anticipate that an increased numbers of authors increases the time it takes to write a paper, including doing the underlying research.

The impact of colons and question marks on citations is less certain. As discussed in the introduction, on the one hand they may increase the attraction factor in particular, but on the other hand, in constraining the title to a certain style, they may at times reduce the informational content. We anticipate that all of the above characteristics and impact will be reflected in the REF and hence can be tested with the REF data base. However, the extent to which they differ between different disciplines will also help inform us about those disciplines, and in some cases the problems of evaluating research across disciplines using the REF methodology. We return to these points in the conclusion.

## The data

We downloaded the data on REF submissions which were in Excel format. In all this gave rise to some 190, 962 outputs to analyse^5^ 155,552 of these were papers in journals. The data was then loaded into Stata where it was analysed. There were 26,719 books or book chapters and the remaining were neither published books nor papers. Books were more common in Panel D submissions, e.g. History. In this paper we focus solely on journal papers. Journal papers were identified as such by their having an ISSN number. The citations data was provided only to a limited number of sub-panels assessing the UoAs. It was obtained from the Scopus database. A final ‘snapshot’ of citation counts was taken shortly after the REF submissions deadline. These were then provided to the relevant sub-panels for use in the assessment.^6^ Title length was based on character length including spaces between words, colons, question marks, etc. An alternative is to use the number of words. In reality there is relatively little difference in the results when we use either of these options and, as would be expected, the correlation between the two is high at 0.965. In the work which follows the use of characters tended to yield slightly more significant results than words, and for this reason we choose to base the measurement of title length on characters. However throughout we indicate when the results significantly differ between these two options.

There are substantial differences between disciplines as can be seen from Table 2. We report the panel medians, rather than the means, as the latter are influenced by outliers of individual UoAs. Thus for Panel A, the title length of 109.85 is the median value of the six UoA means. The journal paper was very much the norm in health and the sciences, with over 99 % of papers in both panels being journal papers. In contrast, journal papers are less common in the social sciences and very much less common in the arts and humanities. The most parsimonious UoAs with respect to title length were philosophy, economics and classics. The least parsimonious were public health, clinical medicine and agriculture. In some cases titles played the role of mini abstracts. Not all disciplines reported citations. Of those that did the most were in clinical medicine, physics and public health. The number of authors differed substantially between disciplines. The fewest tended to be in the arts and humanities (Panel D) and the most in health and the sciences (Panels A and B). The figures are startling and the average for physics of 131.2 is an extreme, heavily affected by one or two outliers. But even so, there are still substantial differences if we take the 75th percentile. Differences between disciplines in the use of colons were also substantial. Clearly academics are not an homogenous group, at least based on this data.

**Fig. 1:**
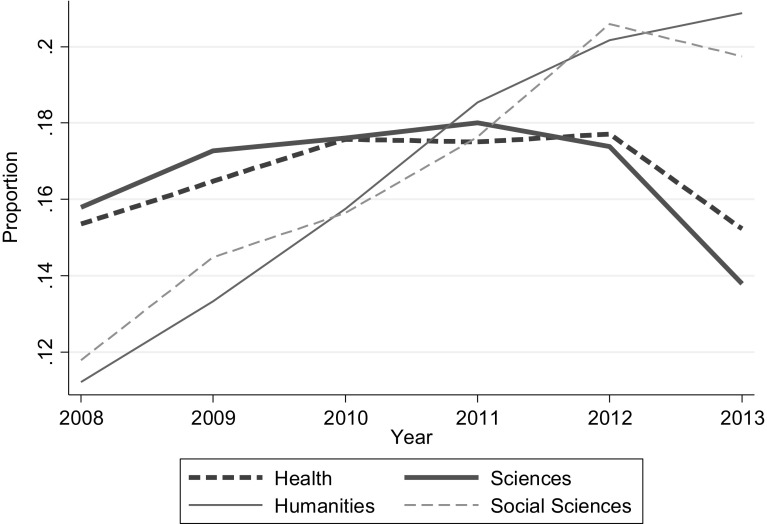
Journal papers’ year of publication

There were also substantial differences in the average publication dates of the papers. The latest were in philosophy, music and classics. The earliest were for physics, earth systems and chemistry. The pattern of dates over time tended to be an inverted U shaped for the sciences and health (Panels A and B) as can be seen from Fig. 1. For the social sciences (Panel C) it peaked in 2012 and for the arts and humanities (Panel D) it carried on increasing throughout the period. The inverted U shaped patterns are suggestive that the REF and other similar such exercises are impacting on the timing of research and research publications, if not we would expect publication dates to be uniformly distributed and most importantly not to be lower in the initial and final years. It is consistent with researchers tending to plan new papers at the beginning of a REF period, with the normal research and publication lags leading to publications 2 or 3 years into the REF cycle. Once researchers having got their four good papers they might tend to reduce their research intensity, or at least delay publication to fit in with the next REF round, as suggested earlier would be the case. That this does not happen in the arts and humanities and social sciences is perhaps suggestive of longer publication lags.

Colons were least common in mathematics, electrical engineering, biological sciences and aeronautical engineering, but could be found in abundance in english language, media studies and social work. There is a similar pattern for question marks. They are virtually absent in Engineering and also Mathematical Sciences, but are particularly prominent in Panel C relating primarily to the social sciences. This is particularly the case for politics, law and social work. We also noted in the introduction that question marks may constrain the style of the title. In support of this, we find that question marks tend to lengthen titles. In 15 of the UoAs, particularly in Panels C and D, the title length was significantly longer with a question mark than without at the 1 % level of significance, and in 22 UoAs at the 5 % level. This was the case whether title length was based on the number of words or characters. In no UoA was the title significantly shorter at the 1 % level and just once at the 5 % level.

We noted in the literature review that there was the possibility that in most disciplines single authors more commonly use colons, for example, in the title than do multiple authors. However, when the number of co-authors is high the result tends to be reversed (Hartley 2007). In Table 3 we break down the variables of interest by categories of authorship numbers. We find some evidence for the tendency noted by Hartley, and in addition evidence from both title length and use of a question mark that for very large author numbers, behaviour tends to resemble that of a small group of authors. This may be because when we have a large numbers of authors, or hyperauthorship, a small number are de facto leaders and, at least with respect to the paper, effectively make the decisions. This is consistent with Beaver’s (2001) observation that having 10–12 individuals working on the same project may be similar to “old style collaboration” if they are viewed as two collective individuals, i.e. from laboratory collectives. However it is also possible that this reflects differences between UoAs rather than the impact of author numbers, with large author numbers and question marks more prevalent in some UoAs than others.

**Table 3:** Summary data by number of authors

Number of authors	Title length	Citations	Observations	Year	Colon %	Question mark %
1	78.4	13.8	25,882	2010.75	55.01	10.04
2–4	87.5	16.4	66,155	2010.58	33.27	6.27
5–9	102.5	23.7	39,469	2010.49	24.87	2.50
10–24	109	42.2	14,990	2010.59	24.32	1.28
25–49	98.6	85.2	1423	2010.74	26.28	0.70
≥49	84.8	78.1	7633	2010.87	40.14	7.62

(i) The average title length in terms of characters, (ii) the average number of citations, (iii) the number of potential observations for each author grouping, (iv) The average year of the publications, (v) the percentage of paper titles with a colon and (vi) the percentage of paper titles with a question mark

In Table 4 we regress title length, citations, year of publication, use of colons and question marks on dummy variables reflecting the number of authors grouped as in Table 3. We also include dummy variables reflecting the UoA, and, for the regressions relating to citations, a dummy variable reflecting the year of publication. The latter is necessary as, for example, a paper published in 2010 stands more chance of having garnered citations than one published in 2013. In all cases the appropriate regression technique was used as indicated in the Table. In addition throughout the paper standard errors have been corrected for heteroscedasticity, and there is just one observation per paper, i.e. multiple submissions of the same paper are included just once. The results largely confirm those of Table 3. Looking first at title length, the signs of the coefficients need to be compared with the default case of 50 or more authors. We can see that single authored papers tend to have a significantly smaller title than the default case. However all other categories of authors have significantly more. The pattern thus suggests that title length steadily increases up to 25–49 authors and then begins to decline.^7^ The coefficients relating to the year of publication again suggest that this peaks at 25–49 authors. Colons are most commonly used by single authors. Finally the regression indicates that the use of question marks steadily declines once we have 5–9 authors, but picks up again for the 50 plus author group. These results relate to all disciplines as a whole. In the next section we analyse the effects on individual UoAs.

**Table 4:** Regression results with number of authors

Number of authors	Title length	Citations	Year	Colon %	Question %
1	−0.0582** (10.13)	−45.6** (11.42)	−0.161** (10.71)	0.122** (6.59)	−0.0293 (1.12)
2–4	0.0154** (2.95)	−42.7** (10.81)	−0.135** (9.49)	−0.0095 (0.54)	0.0245 (0.96)
5–9	0.0889** (16.12)	−36.7** (9.22)	−0.101** (6.66)	−0.0753** (3.89)	−0.243** (8.02)
10–24	0.111** (18.01)	−19.7** (4.76)	−0.00727 (0.42)	−0.0914** (4.01)	−0.447** (10.54)
25–49	0.0258* (2.13)	20.30** (3.31)	0.12** (3.39)	0.0357 (0.74)	−0.65** (4.72)
Observations	139,705	62,878	139,705	139,705	139,705

The independent variables were dummy variables proxying the number of authors. The regressions are: (i) an OLS regression on the log of the title length, (ii) a Tobit regression on the number of citations with a lower bound of zero, (iii) an ordered probit regression on year of publication, (iv) a probit regression on whether a colon is present in the title and (v) a probit regression on whether a ‘?’ is present in the title. All regressions contain dummy variables for different UoAs and a constant term. Standard errors corrected for heteroscedasticty

*^,^ ** denotes significance at the 1/5 % levels

## The regression results for individual UoAs

The regressions which follow focus on individual UoAs, firstly with respect to the impact of the number of authors on a variety of dependent variables relating to the title and the year of publication, and secondly the impact of the title characteristics on subsequent citations. The results from Table 4 suggest that an increasing number of authors impacts on all the dependent variables, although in different ways. In these further regressions we just include the log of authors as this simplifies the analysis and focuses attention on the impact of this single variable and how it differs between UoAs. We log the number of authors as the impact of this variable is unlikely to be linear in any of the regressions. The regression with the length of title as the dependent variable was done by ordinary least squares (OLS). That for the year of publication by ordered probit, and the use of colons and question marks was analysed by binomial probit. In all cases the standard errors have been corrected for heteroscedasticity.

The regression results are shown in Table 5. In column one we look at the year of submission. A positive coefficient means a later submission is associated with an increase in author numbers. The results are particularly striking. In 17 of the UoAs the coefficient on the number of authors is significantly positive. In none is it significantly negative. The results are particularly strong for panels A and B, representing health and the sciences. But it is also evident in panel C, relating to the social sciences, and not found at all in panel D, relating to the arts and humanities. In column two the dependent variable is the title length. This tends to increase with the number of authors, which is positively significant in 34 of the 36 UoAs, generally at the 1 % level, otherwise the 5 % level. The biggest impact is for classics and being as the number of authors are logged, the results indicate that a doubling of the number of authors increases the title length by almost 14 characters. The biggest impacts tend to be in the arts and humanities, but because multiple authorship is so much more common in the sciences and health, the greatest impact of multiple authorship, as such, on title length is likely to be in these disciplines. The results with respect to colons are less clear-cut, although there is a definite pattern. There are 16 significantly negative coefficients and five significantly positive ones. In panels A and B, the health and sciences panels, UoAs tend to have a negative coefficient, but computing, mathematics and aeronautical engineering UoAs have significantly positive ones. In panels C and D the tendency is towards negative coefficients. The impact on question marks is slightly clearer, particularly in panels A and B. In all, 15 of the coefficients are significantly negative, with 10 being in panels A and B. There are just two significantly positive coefficients: in philosophy and economics. To enhance intuition of the impacts, the final three columns show, when significant, the impact of increasing the number of authors from one to four on the publication date and the probabilities of using a colon and a question mark. For example, for Agriculture the predicted publication date increases by 3.43 months, the probability of using a colon declines by 0.061 and using a question mark by 0.07, i.e. 6.1 and 7 % points respectively.

**Table 5:** Coefficients on Log of number of authors

	Year of publication		Title length		Colon used		Question mark used		Increasing authors		
	Co eff.	*t* stat.	Co eff.	*t* stat.	Co eff.	*t* stat.	Co eff.	*t* stat.	Year pub	Colon	Question
Panel A											
Clinical medicine	0.158**	(11.08)	0.542	(1.09)	0.001	(0.06)	−0.267**	(5.29)	5.63	ns	−0.024
Public health	0.049**	(2.63)	7.379**	(9.27)	−0.035	(1.53)	−0.352**	(8.09)	1.81	ns	−0.102
Allied health	0.048**	(2.88)	7.862**	(13.18)	−0.183**	(8.31)	−0.311**	(8.43)	1.78	−0.097	−0.059
Psychology	0.068**	(4.11)	9.100**	(16.55)	−0.140**	(7.06)	−0.370**	(10.89)	2.52	−0.075	−0.087
Biological sci	0.095**	(5.21)	2.934**	(5.45)	−0.214**	(6.82)	−0.417**	(5.69)	3.43	−0.072	−0.043
Agriculture	0.093**	(3.20)	2.186*	(2.39)	−0.146**	(3.51)	−0.444**	(5.14)	3.43	−0.061	−0.070
Panel B											
Earth systems	0.057**	(2.70)	1.426*	(2.03)	−0.125**	(4.50)	−0.197**	(3.73)	2.14	−0.06	−0.029
Chemistry	0.114**	(4.29)	2.502**	(2.91)	−0.113**	(3.40)	−0.156*	(2.00)	4.15	−0.051	−0.009
Physics	0.083**	(9.80)	3.605**	(13.16)	−0.074**	(6.96)	−0.136**	(3.93)	2.78	−0.026	−0.01
Mathematical sci.	−0.047	(1.86)	6.863**	(10.69)	0.182**	(4.28)	0.175	(1.71)	ns	0.041	ns
Comp science	0.076**	(2.97)	7.428**	(11.09)	0.175**	(4.84)	−0.077	(0.82)	2.76	0.06	ns
Aeronautical eng.	0.128**	(4.00)	6.602**	(6.32)	0.116*	(2.32)	0.023	(0.19)	4.68	0.032	ns
Electrical eng.	0.086**	(2.82)	2.644**	(3.00)	0.099	(1.84)	−0.165	(1.07)	3.13	ns	ns
Civil eng.	0.067	(1.22)	3.966*	(2.29)	−0.067	(0.83)	−0.376*	(1.98)	ns	ns	−0.037
General eng.	0.017	(0.91)	5.560**	(8.60)	0.051	(1.88)	0.059	(1.02)	ns	ns	ns
Panel C											
Architecture	0.01	(0.31)	5.113**	(5.11)	−0.333**	(7.79)	−0.261**	(4.29)	ns	−0.182	−0.055
Geography	−0.008	(0.42)	6.842**	(11.09)	−0.393**	(16.72)	−0.167**	(4.82)	ns	−0.214	−0.035
Economics	0.031	(0.60)	3.970**	(2.94)	0.329**	(4.81)	0.266**	(3.03)	ns	0.156	0.061
Business	0.155**	(6.60)	4.626**	(6.96)	0.018	(0.67)	−0.047	(1.28)	5.88	ns	ns
Law	0.120*	(2.26)	7.025**	(4.65)	0.162**	(2.62)	0.097	(1.36)	4.42	0.088	ns
Politics	0.153**	(3.24)	6.517**	(5.26)	0.058	(1.05)	0.122	(1.95)	5.58	ns	ns
Social work	0.099**	(3.47)	10.096**	(10.99)	−0.058	(1.64)	−0.124**	(2.90)	3.59	ns	−0.041
Sociology	0.112*	(2.33)	10.194**	(7.81)	−0.057	(1.00)	0.045	(0.62)	4.41	ns	ns
Anthropology	−0.081	(1.92)	5.342**	(4.39)	−0.415**	(7.64)	−0.006	(0.10)	ns	−0.221	ns
Education	−0.011	(0.38)	6.664**	(7.68)	−0.119**	(3.56)	−0.072	(1.74)	ns	−0.063	ns
Sport science	0.036	(1.09)	13.267**	(12.66)	−0.386**	(8.97)	−0.453**	(6.69)	ns	−0.207	−0.106
Panel D											
Area studies	−0.101	(1.20)	3.466	(1.49)	0.138	(1.31)	0.213	(1.77)	ns	ns	ns
Modern languages	−0.009	(0.19)	4.185**	(2.69)	−0.194**	(3.16)	−0.26*	(2.53)	ns	−0.106	−0.043
English language	−0.016	(0.21)	14.521**	(5.48)	−0.117	(1.21)	0.035	(0.21)	ns	ns	ns
History	0.001	(0.01)	4.507*	(2.04)	0.197	(1.90)	0.162	(1.18)	ns	ns	ns
Classics	−0.045	(0.18)	19.713**	(3.11)	0.144	(0.65)	0.216	(0.83)	ns	ns	ns
Philosophy	0.115	(0.97)	12.270**	(3.33)	0.032	(0.22)	0.432**	(2.64)	ns	ns	0.133
Theology	0.402	(1.74)	17.587*	(2.53)	0.281	(0.94)	−0.161	(0.44)	ns	ns	ns
Arts	0.049	(1.05)	7.951**	(5.79)	−0.432**	(7.73)	−0.026	(0.28)	ns	−0.235	ns
Music	0.155	(1.93)	7.027*	(2.40)	−0.286**	(3.05)	−0.162	(1.04)	ns	−0.154	ns
Media studies	0.001	(0.01)	5.921**	(4.12)	−0.189**	(3.01)	0.061	(0.73)	ns	−0.098	ns

The coefficients and t statistics (in (.)) on the log of the number of authors in (i) an ordered probit regression of year of publication, (ii) an OLS regression on the title length, (iii) a probit regression on whether a colon is present in the title and (iv) a probit regression on whether a ‘?’ is present in the title. All regressions contain dummy variables for different UoAs and a constant term. Standard errors are corrected for heteroscedasticty. The final three columns show, when significant (ns means not significant), the impact of increasing the number of authors from 1 to 4, derived from appropriate regressions. The figure relating to publication date is in months, the other two figures reflect the change in the probability of a colon or question mark

*^,^** denotes significance at the 1/5 % levels

The final set of regressions relate to the various impacts of different title characteristics and the log of the number of authors on the number of citations and are estimated by the Tobit estimator with a lower bound of zero, with standard errors again corrected for heteroscedasticity. We do not have citation data on all UoAs, rather primarily from panels A and B. In addition, sometimes when we are supposed to have the data we get the missing value code. The UoAs with at least 40 observations are shown in Table 6. The most consistent result relates to the number of authors which has a significantly positive coefficient in all twelve UoAs. Use of a colon has a significantly positive impact in five of the UoAs. There is no real hint of negative significance in any UoA, with nothing significant at even the 10 % level of significance. The use of a question mark does however significantly reduce citations in four of the UoAs. There is only one positive coefficient but it is not significant at even the 10 % level. Finally citations significantly decline with title length in ten of the eleven UoAs, with the impact almost significant at the 10 % level in the eleventh.^8^ As an example of the size of impact, doubling the title length and using a question mark reduces the number of citations by 15.9 and 19.0 respectively for Clinical Medicine.^9^ The impact in some other disciplines is much less, but so too are the average number of citations as shown in Table 2. We also show the results of estimating a single equation based on all available citation data including dummy variables for different UoAs. The dependent variable is the ratio of citations to the UoA mean, rather than the number of citations per se, as the latter implies a common absolute impact of, for example, colon use across UoAs with widely differing average citations. Our approach simply implies a common proportionate impact. However, if we had used the number of citations instead of the ratio nothing would have changed in terms of significance. Again the Tobit estimator is used with a lower bound of zero and standard errors corrected for heteroscedasticty. The number of authors, use of colons and question marks and length of title are all significant at the 1 % level, with title length and use of a question mark reducing citations.

**Table 6:** Regressions on citations

	Number of authors	Colon	Question mark	Title length	*F*	Observations
Panel A						
Clinical medicine	30.923** (13.13)	10.833** (6.29)	−15.892** (3.74)	−27.438** (10.00)	101.92	11,945
Public health	30.955** (10.90)	0.689 (0.28)	−11.045** (3.26)	−22.551** (5.48)	35.75	4262
Allied health	14.294** (9.44)	−0.552 (0.78)	−3.939** (3.28)	−7.421** (5.69)	69.63	9404
Psychology	22.175** (11.20)	1.655 (1.59)	−0.754 (0.42)	−8.346** (4.87)	95.98	7984
Biological sci	21.873** (10.21)	8.066** (2.64)	−3.432 (0.99)	−29.908** (11.06)	81.12	7869
Agriculture	17.813** (5.75)	3.329* (2.51)	−5.025 (1.91)	−6.788** (4.17)	24.49	3677
Panel B						
Earth sys	15.861** (5.26)	0.189 (0.14)	−5.169 (1.85)	−18.051** (7.18)	43.82	4700
Chemistry	8.921** (3.42)	−1.786 (1.42)	−3.740 (1.10)	−5.709** (3.47)	73.18	4352
Physics	7.442** (7.04)	17.035** (2.80)	−5.843 (1.26)	−17.821** (6.55)	27.62	5538
Comp sci	10.037** (6.69)	2.044 (1.63)	−2.206 (0.88)	−1.963 (1.34)	33.22	5158
Panel C						
Geography	19.387* (2.19)	−2.789 (0.67)	0.895 (0.15)	−14.071* (2.44)	2.137	46
Economics	5.321** (5.20)	2.739** (3.15)	2.024 (1.49)	−4.174** (3.28)	22.05	1988
All panels	0.505** (24.04)	0.106** (5.00)	−0.169** (4.78)	−0.452**	143.09 (16.19)	62,235

The coefficients, t statistics in (.) and *F* statistic on number of citations in a Tobit regression with a lower bound of zero. Standard errors corrected for heteroscedasticty. The final row relates to a fixed effects regression on all available data in all UoAs, where the dependent variable is the ratio of citations to the UoA mean

*^,^** denotes significance at the 1/5 % levels

## Conclusions

The literature suggests that multiple authorship may bring specialisation gains, with academics with differing expertise combining together to do research they would find difficult to do alone. There are also potential gains from having >1 person looking and thinking about the paper and the resulting communication between individuals. But the literature also suggests that this all comes at a cost, a cost in coordinating research which plausibly increases with the number of cooperating individuals, i.e. authors. The difficulties in reaching agreement between different authors can to an extent be resolved by doing what all want, i.e. expanding the title to reflect everyone’s views. With respect to colons, the co-ordination problem is effectively exacerbated as there are two sub-titles to agree on. Given this, it is expected that we observe that the use of semi colons tends to decline with the number of authors. Titles in the form of questions tend to constrain the title to a particular style, in the process tending to lengthen the title, and thus again may be more difficult to agree upon. Hence once more, we would expect their use to decline as the number of authors increase.

The results tend to be consistent with these expectations and it is clear that multiple authorship leaves a footprint on the style at least of the title, and thus also perhaps the style of the paper itself.^10^ To an extent our results are consistent with previous research. But our results are derived from an analysis across the disciplines, rather than from a specific group of disciplines. They also help explain some of the disagreements between previous research findings, which are in part due to their being some differences between disciplines. The positive impact of the number of authors on title length is evident in most UoAs across all panels. The negative impact of using colons and question marks is less consistent. For the former there are 16 significant negative impacts and five positive ones. For question marks the corresponding figures are 15 and 2 respectively. A negative impact is particularly common in the sciences and the health panels. But the fact that there are differences between disciplines emphasises that we need to be careful about generalising to the whole of scientific work the results from a partial analysis. However, broadly speaking, we can say that the sciences, excluding engineering and mathematics, tend to be characterised by a positive impact of increasing authorship on title length and publication date, and a negative impact on the use of colons and question marks. The UoAs covered by panel D, which we collectively term the arts and humanities, tend to have different characteristics such as smaller titles, fewer authors and more books. There is also much less impact in these disciplines of the number of authors on a paper’s publication date and on the use of colons and question marks. The social sciences, as covered by Panel C, tend to lie between the sciences and the arts and humanities in these respects.

It seems possible that some of these differences can be put down to a combination of two reasons. Firstly difference in discipline culture and secondly the number of authors. Large numbers of authors characterise Panels A and B as can be seen in Table 2, are far less common in Panel C, relating to the social sciences, and even less common in Panel D relating to the arts and humanities. Thus it may be that the relative lack of impact of the number of authors on title characteristics in Panels C and D is in part because the number of authors varies over a smaller range than in Panels A and B. The possibility that differences are culture based raises the question of whether this is because the academics in the different disciplines are different, the disciplines are different, or because of both of these possibilities. There may well be other differences in style, for example with respect to the use of allusions or famous quotations in titles, perhaps separated by colons. These would be difficult to identify using program commands, but future research might do so by visually inspecting each title.

The results suggest that the title characteristics of papers with a very large number of authors are in many respects similar to those with fewer numbers, and we note again that this suggests that such papers have a relatively small number of defacto leaders making decisions on the paper. If this is the case, should they be identified on the paper and differentiated from other authors? Indeed it may be, as suggested by Cronin (2001), that not all authors contribute to the writing of the paper rather than the research. This problem of differential contribution, together with the very large number of authors on some papers does raise questions for exercises such as the REF itself. In particular is it reasonable to give the same weight to a paper with more than a hundred authors as is given to a paper with just one author?

The positive impact of author numbers on publication dates suggests two hypotheses: Firstly that the time it takes to do the research and write and publish a paper increases with the number of authors. But this by itself cannot explain what we have observed. For that to be the case we also need the research to be starting at approximately the same time. If starting points were random then we might also expect publication dates to be random. This is then suggestive that the REF is impacting on the timing of research which is also consistent with the pattern in the publication dates as illustrated in Fig. 1. Of course, other factors unique to the individual authors, and also more general political and economic factors, may impact on the timing of publications. In addition, there will be some authors who are confident that they will have more than enough good publications and for whom considerations of the REF are relatively a minor consideration. But this will not be true of all academics, and if all academics were just ‘doing their best’ and ignoring the REF, we would expect to see a more even flow of publications rather than the inverted U shape for Panels A and B or even the more steady increase characterising Panel D.^11^ Of course this is just one piece of research, albeit one which is consistent with other research such as Hodder and Hodder (2010). But the results need further collaboration by future studies in different contexts. Nonetheless if substantiated, is it disturbing that effectively academics are timing, and perhaps delaying their research, so as to fit in with the REF cycle, particularly if this relates to what is likely to be the best and perhaps most important examples of their research?

## Footnotes

1 There are at least two aspects to attractiveness. The first is the ability of the title to attract attention through, for example, a distinctive title, use of a colon or a question based format. The second, is the extent to which it facilitates the acquisition of information about the paper. Too long a title, may reduce the latter. Indeed from one respect although increasing the amount of information provided, a very long title can be viewed as reducing the information the typical reader absorbs. This will be the case if, by being too long, it deters academics from reading it in full.

2
http://www.ref.ac.uk/.

3 The percentages relate to 2005.

4
http://www.nadinemuller.org.uk/guides-to-academia/the-ref/.

5 This differs slightly from the 191,150 figure on the home page of the REF website.

6 See http://www.ref.ac.uk/about/guidance/citationdata/.

7 If we had used words rather than characters to measure title length, then the coefficients on 2–4 authors and 25–49 authors are insignificant at the 5 % level of significance.

8 Using words rather than characters to measure title length changed relatively little. For Physics, colon usage is significant at the 5 % level rather than 1 %, for Geography the coefficient on title length is not significant at the 5 % level and for Economics, title length was significant at the 5 % level, rather than the 1 % level.

9 The numbers are large, but are reflected in the raw data. For example, Clinical Medicine papers with a title length <90 characters received an average of 52 citations, compared to 31.8 citations for those with >90 characters. The impact of title length is found by multiplying the coefficient by the difference of the log of 4 and the log of 2.

10 Thus, for example Persson et al. (2004) find the number of references to increase with the number of authors, which of course has implications for the text of the paper itself.

11 It is also possible that any drop off in the number of papers for 2013 were affected by the REF submissions having to be submitted by the end of November 2013, but this had little impact on panels C and D.
